# Chemical composition and antibacterial activity of bee venom against multi-drug resistant pathogens

**DOI:** 10.4102/ojvr.v90i1.2097

**Published:** 2023-07-20

**Authors:** Tülin G. Gökmen, Hatice Yazgan, Yıldız Özdemir, Sedat Sevin, Nevin Turut, Şifa Karahan, Funda Eşki, İbrahim Kıvrak, Osman Sezer, Armağan E. Ütük

**Affiliations:** 1 Department of Microbiology, Ceyhan Veterinary Faculty, Cukurova University, Adana, Turkey; 2 Department of Food Hygiene and Technology, Ceyhan Veterinary Faculty, Cukurova University, Adana, Turkey; 3 Microbiology Laboratory, Adana City Hospital, Adana, Turkey; 4 Department of Pharmacology and Toxicology, Veterinary Faculty, Ankara University, Ankara, Turkey; 5 Microbiology Laboratory, Veterinary Control Institute, Adana, Turkey; 6 Department of Obstetrics and Gynecology, Veterinary Faculty, Cukurova University, Adana, Turkey; 7 Department of Chemistry and Chemical Processing Technologies/Cosmetic Technology Program, Muğla Vocational School, Muğla Sıtkı Koçman University, Muğla, Turkey; 8 Parasitology Laboratory, Veterinary Control Institute, Adana, Turkey; 9 Department of Parasitology, Ceyhan Veterinary Faculty, Cukurova University, Adana, Turkey

**Keywords:** bee venom, multi-drug resistant pathogens, antibacterial activity, microdilution method, bioproduct

## Abstract

Bee venom with an antimicrobial effect is a powerful natural product. One of the most important areas where new antimicrobials are needed is in the prevention and control of multi-drug resistant pathogens. Today, antibacterial products used to treat multi-drug resistant pathogen infections in hospitals and healthcare facilities are insufficient to prevent colonisation and spread, and new products are needed. The aim of the study is to investigate the antibacterial effect of the bee venom (BV), a natural substance, on the species of Methicillin resistant *Staphylococcus aureus*, Vancomycin resistant *Enterococcus faecalis*, Carbapenem resistant *Escherichia coli*, Carbapenem resistant *Klebsiella pneumoniae* and Carbapenem resistant *Acinetobacter baumannii*. As a result of this study, it was found that MIC_90_ and MBC_90_ values ranged from 6.25 μg/mL – 12.5 μg/mL and numbers of bacteria decreased by 4–6 logs within 1–24 h for multi-drug resistant pathogens. In particular, Vancomycin resistant *Enterococcus faecalis* isolate decreased 6 log cfu/mL at 50 μg/mL and 100 μg/mL concentrations in the first hour. The effective bacterial inhibition rate of bee venom suggests that it could be a potential antibacterial agent for multi-drug resistant pathogens.

**Contribution:**

The treatment options of antibiotic-resistant pathogens are a major problem in both veterinary and human medicine fields. We have detected a high antibacterial effect against these agents in this bee venom study, which is a natural product. Apitherapy is a fashionable treatment method all over the world and is used in many areas of health. Bee venom is also a product that can be used as a drug or disinfectant raw material and can fill the natural product gap that can be used against resistant bacteria.

## Introduction

Multi-drug resistant (MDR) pathogens are an important global problem in the world. They seriously threaten public health in the basic areas such as food and health sector. Generally, the environment in which MDR pathogens originates are hospitals and cause most health-associated infections (Allegranzi & Pittet 2007). The Centers for Disease Control and Prevention (CDC) estimate that pathogens have affected 1.7 million people in the United States (US) and have resulted in the death of 99 000 people (Haque et al. 2018). Although protection and control programmes are used against these infections, the effect is not sufficient. The increase of MDR pathogens complicates the control of infections, because of the irrational use of antibiotics, the decrease in the effectiveness of last option antibiotics in the treatment, and increased resistance to disinfectants (Machowska & Stålsby Lundborg 2018). For this reason, natural products, that have not previously been in contact with MDR pathogens and have no potential for resistance development, can be an important antibacterial option. To date, many natural antibacterial products have been tested for MDR pathogens, and even various bee products have been tried (Dinkoy 2017). However, there is a very limited number of studies on bee venom (BV); specifically on the most common and important species such as Meticillin resistant *Staphylococcus aureus* (MRSA), Vancomycin resistant *Enterococcus faecalis* (VRE), Carbapenem resistant *Escherichia coli, Klebsiella pneumoniae*, and *Acinetobacter baumannii*. Bee venom is an antibacterial mixture against gram-positive and negative bacteria and contains various peptides, amines, phospholipids, volatile compounds, aminocytes, sugars and enzymes (Carpena et al. 2020). In particular, melittin, apamin and phospholipase C are the most important components. The fact that BV contains active ingredient that causes cell membrane pore formation and membrane phospholipid destruction makes it an important antibacterial bioproduct (Funayama et al. 2012).

In this study, the aim was to evaluate the antibacterial activity of BV; a natural product, as an option to be used for the control of MDR gram-positive and gram-negative pathogens, which are the biggest problem in terms of morbidity, mortality and economic aspects. To fulfil this, minimal inhibition concentration (MIC) and minimal bactericidal concentration (MBC) as well as the chemical composition of honey BV were assessed.

## Research methods and design

### Collection of bee venom

Samples of BV were collected by the BV collector (Beesas Ar, Turkey) from *Apis mellifera anatoliaca* during the citrus honey season between May 2021 and June 2021. After collection, the BV was scraped with a scalpel on glass plates and stored under room conditions for 8 h and placed in the freezer at −18 °C (Gökmen et al. 2023).

### Determination of content and compositions of bee venom

The amount of apamin, phospholipase-A2 and melittin components in BV was analysed by high-performance liquid chromatography (HPLC) variable wavelength detector (VWD) (Agilent 1260 Series). Infinitylab Poroshell C18 EC-C18 (4.6 mm × 50 mm, 2.7 micron) column was used for separation. While the optimum separation temperature was 20 °C, the column flow rate was 1 mL/min. Apamin (Sigma-A1289), Phospholipase A2 (Sigma-P9279) and Melittin (Sigma M2272) standard solutions were prepared at 10 μg/mL, 20 μg/mL, 50 μg/mL and 100 μg/mL concentrations. The buffer A (0.1% trifluoroacetic acid [TFA] in water [H_2_O]) and the buffer B (0.1% TFA in acetonitril) were used as mobile phases. The Gradian programme has been optimised for peak holding times. Absorbance measurements were made at 218 nm (Gokmen et al. 2023).

### Bacterial strains

In the current study, Meticillin resistant *S. aureus* (MRSA), Vancomycin resistant *E. faecalis* (VRE), Carbapenem resistant *E. coli* (CREC), Carbapenem resistant *K. pneumoniae* (CRKP) and Carbapenem resistant *A. baumannii* (CRAB) strains were used from the Cukurova University, Ceyhan Veterinary Faculty, Department of Microbiology (Adana, Turkey). multi-drug resistant pathogens were identified by VITEK^®^2 Identification System (Biomerieux).

### Detection of antibiotic resistant by disc diffusion method

**FIGURE 1:**
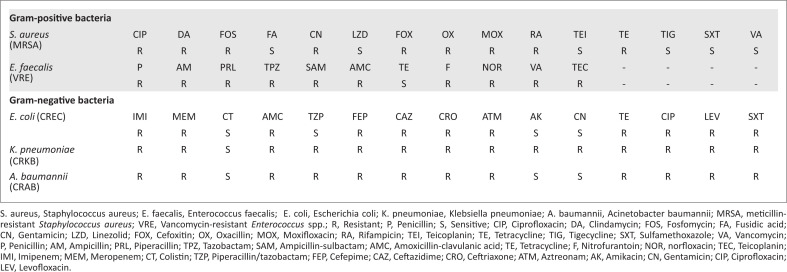
Kirby-Bauer disc diffusion test for multi-drug resistant pathogens.

Kirby-Bauer disk diffusion method was used to determine the antibiotic sensitivity profiles of MDR pathogens (Bauer et al. 1966). Various discs applied in all species-specific routine antibiograms were used. Antibiotic resistance profiles are shown in Figure 1.

### Determination of minimal inhibition concentration and minimal bactericidal concentration value of bee venom

Bee venom MIC and MBC were detected by using a microdilution method (CLSI 2015). The concentrations of 6.25 μg/mL, 12.5 μg/mL, 25 μg/mL, 50 μg/mL, 100 μg/mL, 200 μg/mL, 400 μg/mL and 800 μg/mL were prepared and added to the wells in the microplates with the Muller Hinton broth (MHB) and BV. For the five MDR pathogens, in each well, bacterial density was set to 2 cfu/mL × 106 cfu/mL. Wells containing MHB and bacterial suspension were used as positive control, only BV and MHB wells were used as negative control. The absorbance of the microplate, which was incubated at 37 °C during the night, was read at 560 nm and 620 nm. According to the absorbance values on microplate, the MIC value was determined for each MDR pathogen. Later, MBC values were determined by overcoming the samples in the microplate to the Müller–Hinton agar (MHA).

### Time-kill assays

The time-kill assay of BV against all tested bacteria was evaluated at their MIC according to the previous method with minor modifications (Chuesian et al. 2019). Different time intervals (0 h, 1 h, 3 h, 6 h, 12 h and 24 h) were applied in the assay. Multi-drug resistant pathogens suspension at 0.5 McFarland turbidity (1.5 cfu/mL × 10^8^ cfu/mL) was inoculated in mediums containing MIC value and the previous and next concentrations of the detected MIC value. The bacteria-BV mixture was incubated for 0 h, 1 h, 3 h, 6 h, 12 h and 24 h at 37 °C. Point one mililitre of each bacterial suspension was spread on MHA agar plate at each time point and incubated at 37 °C for 24 h. After incubation bacterial colonies were counted for time-kill assay curve.

### Ethical considerations

The study was approved on 04 April 2022 by the Ethics Committee of Adana Veterinary Control Institute, Adana, Turkey (approval no: 04/04/2022-1/227).

## Results and discussion

**FIGURE 2:**
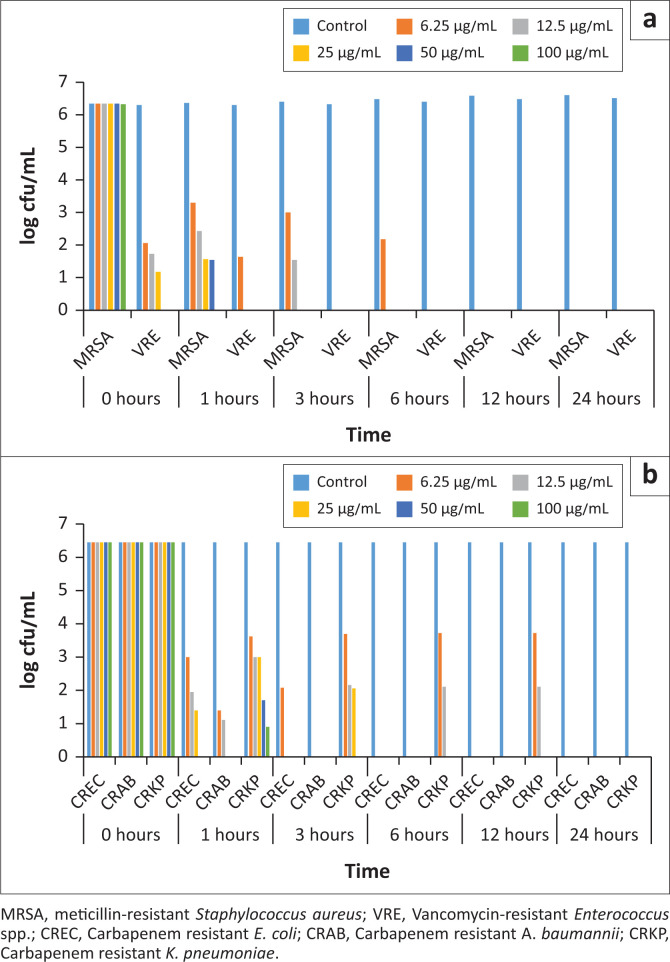
Time-killing assay graphics for multi-drug resistant pathogens (a) gram-positive multi-drug resistance bacteria, (b) gram-negative multi-drug resistance bacteria.

Bee venom consists of 88% water, and in the dry matter there are various peptides such as apamin, histamine, hyaluronidase, phospholipase A (PLA) (Wehbe et al. 2019). It is known that the composition of BV contains about 18 different bioactive substances (Carpena et al. 2020). In this study, the main compounds detected in BV were melittin and PLA at proportions of 70.49% and 13.51%, respectively, and the other compound was apamin at 3.85%. Although BV has different ingredients, the high amount of some substances may be very meaningful in terms of antimicrobial activity. Mellittin causes pore in the membrane at levels of 50% and 60% (Sonmez et al. 2022; Tanuğur-Samancı & Kekeçoğlu 2019). In addition, the proportion of PLA, which ensures the destruction of phospholipids in the membrane structure, is between 10.00% and 20.95% in a qualified BV (Tanuğur-Samancı & Kekeçoğlu 2019). Higher concentrations of PLA and melittin in the current study may be a good indicator for a high quality BV (Figure 2, Table 1).

**TABLE 1:** The concentrations of components of bee venom (±s.d.).

Sample	Apamin (%)	Phospholipase A2 (%)	Melittin (%)
Bee venom	5.17 ± 0.18	14.62 ± 0.20	76.89 ± 0.20

s.d., standard deviation.

In this study, the authors aimed to determine the antibacterial activity of BV on the isolates that we identified as MDR by Kirby Bauer disc diffusion method. Therefore, we used the microdilution method and determined MIC and MBC values for MDR pathogens. Microdilution method was applied to the microplates using 6.25 μg/mL, 12.5 μg/mL, 25 μg/mL, 50 μg/mL, 100 μg/mL, 200 μg/mL, 400 μg/mL and 800 μg/mL concentrations of BV. Absorbances at 560 nm and 620 nm wavelengths were measured with an ELISA reader. It was determined that the MIC_90_ and MBC_90_ values of the pathogens varied between 6.25 μg/mL and 12.5 μg/mL (Table 2).

**TABLE 2:** Microdilution and time-killing assay at various concentrations for multi-drug resistant pathogens.

	MIC value	MBC value	Time-killing	
			Value	Times
**Gram-positive bacteria**				
*S. aureus* (MRSA)	6.25 μg/mL	6.25 μg/mL	6.25 μg/mL	12 h
			12.5 μg/mL	6 h
			25 μg/mL	3 h
			50 μg/mL	1 h
*E. faecalis* (VRE)	6.25 μg/mL	6.25 μg/mL	6.25 μg/mL	3 h
			12.5 μg/mL	1 h
			25 μg/mL	1 h
			50 μg/mL	0 h
**Gram negative bacteria**				
*E. coli* (CREC)	6.25 μg/mL	6.25 μg/mL	6.25 μg/mL	6 h
			12.5 μg/mL	1 h
			25 μg/mL	1 h
			50 μg/mL	1 h
*K. pneumoniae* (CRKP)	12.5 μg/mL	12.5 μg/mL	6.25 μg/mL	Growth
			12.5 μg/mL	12 h
			25 μg/mL	6 h
			50 μg/mL	3 h
*A. baumannii* (CRAB)	6.25 μg/mL	6.25 μg/mL	6.25 μg/mL	3 h
			12.5 μg/mL	1 h
			25 μg/mL	1 h
			50 μg/mL	1 h

MRSA, meticillin-resistant *Staphylococcus aureus*; MIC, minimal inhibition concentration; MBC, minimal bactericidal concentration; VRE, Vancomycin-resistant *Enterococcus* spp.; CREC, Carbapenem resistant *E. coli*; CRAB, Carbapenem resistant *A. baumannii*; CRKP, Carbapenem resistant *K. pneumoniae*.

In the current study, we evaluated two species MDR gram-positive bacteria, which are important problems in healthcare services. In a previous study, MIC and MBC values of BV were determined as 0.085 μg/mL and 0.11 μg/mL, and 0.10 μg/mL and 0.14 μg/mL, respectively (Han et al. 2016). Kong et al. 2020 determined the MIC value of BV as 15.6 μg/mL for MRSA. The MIC values of melittin against MRSA, were between 0.125 μg/mL and 32 μg/mL (Choi et al. 2015; Lima et al. 2021; Marques Pereira et al. 2020). Also, we determined the MIC and MBC values as 6.25 μg/mL against VRE isolate. Al-Ani et al. (2015) reported that the MIC values of BV and mellitin were 100 μg/mL and 500 μg/mL, respectively.

The authors evaluated the MIC and MBC values of three MDR gram-negative bacteria. Carbapenem resistant *A. baumannii* is a very persistent bacteris found in intensive care units. Minimal inhibition concentration and MBC values were observed as 6.25 μg/mL for BV in this study. In various studies, the MIC value of BV was determined as 31.25 mg/mL and the MIC values of melittin was 0.5 μg/mL – 1 μg/mL and 8 μg/mL – 16 μg/mL (Al-Safar et al. 2018; Askari et al. 2021; Bardbari et al. 2018). Carbapenem resistant *K. pneumoniae* causes pneumonia and urinary tract infections with high morbidity and mortality in hospitals. The authors determined MIC and MBC values as 12.5 μg/mL for BV. The MIC value of melittin was reported as 32 μg/mL for *K. pneumoniae* carrying the KPC gene (Askari et al. 2021). Also, *E. coli* is one of the most important pathogens in sepsis. In the current study, MIC and MBC values were determined as 6.25 μg/mL for BV. However, there is no study on the antibacterial activity of whole BV on Carbapenem resistant *K. pneumoniae and E. coli.*

In the time-kill analysis, inhibition time was investigated at different concentrations of BV. Multi-drug resistant isolates which reproduced in MBH containing 6.25 μg/mL, 12.5 μg/mL, 25 μg/mL and 50 μg/mL BV were inoculated and evaluated at MHA 0 h, 1 h, 3 h, 6 h, 12 h and 24 h. It was observed that the time killing periods varied between 1 h and 24 h and the concentration were ranging between 6.25 μg/mL and 12.5 μg/mL (Table 2, Figure 2).

The time-killing assay was applied to all bacteria. Bacteria were grouped as gram-positive and gram-negative. The MIC and MBC values for MRSA and VRE isolates were determined as 6.25 μg/mL against gram-positive bacteria. The inhibition time point of MRSA isolate was 12 h at 6.25 μg/mL. It was determined that *S. aureus* USA300 reduced 8 log cfu/mL at 100 μg/mL BV concentration in 1 h (Choi et al. 2015). Han et al. (2016) reported the, MIC values for two MRSA isolates as 0.17 μg/mL and 0.85 μg/mL and the amount of MRSA decreased by 3 log cfu/mL. Similarly, to the current study, BV reduced the amount of MRSA by 6 log cfu/mL for 100 μg/mL concentration at 1 h. Inhibition time point of VRE isolate was 3 h at 6.25 μg/mL. Interestingly, in the current study, as soon as the VRE contacted BV at 50 μg/mL and 100 μg/mL, the growth curve decreased by 6 log cfu/mL in 0 h. The decrease of 6 log cfu/mL in 0 h was an indication that VRE was inhibited as soon as it contacted with BV. To the best of the authors’ knowledge this is the first study about the application of whole BV time-killing assay to VRE isolate. The inhibition time points at all BV concentrations for VRE isolate were shorter than MRSA isolate.

The MIC and MBC values for Carbepenem resistant *E. coli* and *A. baumannii* isolates were determined as 6.25 μg/mL against gram-negative bacteria. The inhibition time points of Carbapenem resistant *A. baumannii* and *E. coli* were 3 h and 6 h, at the concentration of 6.25 μg/mL. The inhibition times of these two pathogens were 1 h at concentrations of 12.5 μg/mL, 25 μg/mL and 50 μg/mL. The MIC and MBC values were 12.5 μg/mL for Carbapenem resistant *K. pneumoniae*. The inhibition times of BV were determined as 12 h, 6 h and 3 h, respectively at the concentration of 12.5 μg/mL, 25 μg/mL and 50 μg/mL (Figure 2).

Carbapenem resistant *K. pneumoniae*, had higher MIC value of BV (12.5 μg/mL) and inhibition time (24 h) in the time-killing assay. Also, it was inhibited at the concentrations of 50 μg/mL and 100 μg/mL in 3 h.

As a result, BV showed a strong antibacterial activity against MDR pathogens, which are difficult to treat and eradicate in areas such as hospitals, veterinary clinics and food sectors. Despite this powerful antibacterial activity, it is a natural product that should be approached carefully in human and animals as it can cause allergic reactions. Bee venom can be used as a disinfectant in the human and animal hospitals, veterinary clinics, dental clinics, nursing homes and food industry. However, more studies need to be done for determining its disinfecting dose.
